# Phylogenomic analyses support a new subgenus, *Rojovitis*, of the grape genus *Vitis* from Mexico

**DOI:** 10.3389/fpls.2025.1580648

**Published:** 2025-05-19

**Authors:** Jun Wen, Angélica Quintanar Castillo, Marcelo R. Pace, Alicia Talavera, Luke Sparreo, Gabriel Johnson, Gary A. Krupnick, Ze-Long Nie

**Affiliations:** ^1^ Smithsonian Institution, Department of Botany, National Museum of Natural History, Washington, DC, United States; ^2^ Posgrado en Ciencias Biológicas, Instituto de Biología, Universidad Nacional Autónoma de México, Mexico City, Mexico; ^3^ Departamento de Botánica y Herbario Nacional de México, Instituto de Biología, Universidad Nacional Autónoma de México, Ciudad de México, Mexico; ^4^ Center for Biodiversity and Evolution, New York Botanical Garden, Bronx, NY, United States; ^5^ Hunan Provincial Key Laboratory of Ecological Conservation and Sustainable Utilization of Wulingshan Resources, College of Biology and Environmental Sciences, Jishou University, Jishou, Hunan, China

**Keywords:** grapes, hybridization, IUCN, new subgenus, phylogenomics, resources, Vitaceae, *Vitis*

## Abstract

Despite the tremendous economic significance of grapes, the systematics of the grape genus remains understudied. Based on recent fieldwork, phylogenomic analyses using both nuclear and plastid genomes, as well as morphological comparisons, we report a new grape subgenus, *Rojovitis*, endemic to Mexico. The new subgenus constitutes a clade that diverged early in the evolutionary history of *Vitis*, yet there is cytonuclear discordance in its position, suggesting hybridization is a likely mechanism in its origin. Subgenus *Rojovitis* contains two species, *Vitis martineziana* J. Wen from Chiapas and *V. rubriflora* J. Wen from Jalisco, both new to science. In comparison to the two other subgenera of the grape genus (subgenus *Vitis* and subgenus *Muscadinia*), *Rojovitis* is characterized by its red flowers and stems with prominent lenticels. The discovery of the third subgenus in *Vitis*, nearly a century after the recognition of the second subgenus, *Muscadinia*, in 1927, represents a major milestone in the systematic research of grapes and their wild relatives. We also use fieldwork and herbarium data to provide distribution maps and conservation assessments of *V. martineziana* and *V. rubriflora* based on IUCN criteria. Both species are assessed to be critically endangered. These findings highlight Mexico as an important region for wild grape resources. The study also demonstrates that biodiversity discovery is far from complete today and that field exploration remains critical for biodiversity science and conservation. These newly discovered resources may benefit humanity, yet these species urgently need to be protected and properly managed due to extensive habitat loss and alteration.

## 1 Introduction

The grape genus *Vitis* is well known for being one of the earliest domesticated woody crops, and grapes have been considered the most economically important fruit crop in the world (Myles et al., 2011; Gerrath et al., 2015; Zhou et al., 2017). The genus is estimated to contain c. 80 species, with at least 45 species in eastern Asia to the Himalayan region, one species in Europe and western Asia, and c. 35 species in North America to northern South America (including approximately 15 species in Mexico). Two subgenera, *Muscadinia* and *Vitis*, have been traditionally recognized based on morphological, anatomical, cytological, and molecular evidence for nearly a century (Rehder, 1927; Moore, 1991; Ma et al., 2018; Wen et al., 2018b; Nie et al., 2023). North America (including Mexico) and eastern Asia represent two major centers of distribution of *Vitis* (Chen et al., 2007; Moore and Wen, 2016), while Europe contains only the economically most important wine grape species, *Vitis vinifera* L., which extends to western Asia (Wen, 2007).

Phylogenetic analyses on the grape genus have supported two major clades within the genus, corresponding to the two subgenera, subgenus *Vitis* and subgenus *Muscadinia* (Ma et al., 2018; Nie et al., 2023). Phylogenetic studies on *Vitis* have resolved subgenus *Vitis* into two main clades corresponding geographically to Eurasia and the New World, especially using chloroplast DNA (e.g., Tröndle et al., 2010; Péros et al., 2011; Zecca et al., 2012; Wen et al., 2018a) and sometimes based on nuclear data (Miller et al., 2013; Wan et al., 2013; Ma et al., 2018). Recent studies employing nuclear phylogenomic data have suggested paraphyly of the New World *Vitis* subgenus *Vitis*, with the Asian taxa nested within a New World grade, even though the plastid genome data supported a major North American clade sister to a Eurasian clade (Nie et al., 2023; Talavera et al., 2023). The observed cytonuclear discordance was suggested to be caused by deep hybridization events (Nie et al., 2023). Several studies have revealed extensive hybridizations within subgenus *Vitis* (Aradhya et al., 2013; Ma et al., 2023; Nie et al., 2023).

Despite the long historical interest in *Vitis* (Planchon, 1887; Munson, 1909), there are still taxonomic and phylogenetic gaps, with Mexico and China remaining poorly explored (Ma et al., 2023; Nie et al., 2023). We recently conducted extensive field and herbarium work throughout Mexico, a region especially rich in early-diverged lineages of *Vitis* as well as its close allies currently placed in *Ampelocissus*. This study aims to assess the phylogenetic and taxonomic positions of several morphologically unique collections recently made in Mexico. We performed phylogenetic analyses of the grape genus *Vitis* and its close allies in *Ampelocissus*, employing 1,013 nuclear genes targeted by a set of Vitaceae baits (Talavera et al., 2023) and using plastid genomes (plastomes) (Wen et al., 2018a).

## 2 Materials and methods

### 2.1 Taxon sampling, DNA extraction, sequencing, and data assembly

We sampled 60 accessions, including 51 *Vitis* samples representing its taxonomic and geographic diversity (45 of the 80 *Vitis* species, or 56.25% species coverage from all geographic regions), eight accessions of the close relative *Ampelocissus*, and one accession of *Parthenocissus quinquefolius* (outgroup) (**Supplementary Table S1**). Most of the study samples were newly generated, with only a few samples from published papers (Ma et al., 2018, 2021; Talavera et al., 2023). Voucher specimens have been deposited in the United States National Herbarium (US), the Smithsonian Institution, Washington, DC, USA.

DNA extractions were done following a modified SDS method (Johnson et al., 2023). DNAs were quantified with a Qubit 4.0 fluorometer (Thermo Fisher Scientific, Waltham, MA, United States) using a high-sensitivity dsDNA kit and then sheared to a target size of ca. 300–500 bp by sonication (QSonica Q800R3, Newtown, CT, United States). DNA libraries were generated with the KAPA DNA library preparation kit following the manufacturer’s protocol. We pooled six indexed libraries in one reaction with equimolar amounts of 100 ng. Solution-based hybridization and enrichment were carried out using a custom-designed Vitaceae bait set covering 1,013 genes (Talavera et al., 2023). About 40% of the unenriched libraries were added into the target-enriched libraries to recover the plastid genome sequences as by-products. Pooled libraries were sequenced on an Illumina Nova-Seq 6000 platform at Novogene, Sacramento, CA, USA, with paired-end 2 × 150 bp.

Raw reads were quality-filtered using Trimmomatic version 0.39 (Bolger et al., 2014) with a 4-bp-wide sliding window. We used the HybPiper pipeline version 1.3.1 (Johnson et al., 2016) to extract target sequences and remove paralog sequences. For the chloroplast assemblies, we used the complete plastid genome of *Vitis vinifera* (NC_007957) (Jansen et al., 2006) as a reference and assembled all 130 plastid genes using the HybPiper pipeline as described above for the targeted nuclear genes.

### 2.2 Phylogenetic analyses

Target gene sequences were assembled and aligned using MAFFT version 7.407 (Katoh and Standley, 2013). The gene sequences were trimmed with trimAl (Capella-Gutiérrez et al., 2009), removing bases present in less than 25% of the accessions. Based on the complete dataset of 986 loci without paralogs, all matrices were combined into a single supermatrix for phylogenetic inference. Maximum likelihood (ML) analysis was performed in RAxML version 8.2.12 (Stamatakis, 2014) using the GTR substitution model with the CAT approximation of rate heterogeneity during tree search, and the best‐scoring tree from all searches was chosen. Branch support was estimated using a rapid bootstrap algorithm, with the number of replicates determined by the bootstopping criterion.

Gene trees for each locus were also reconstructed separately using RAxML version 8.2.12 with the GTRGAMMA model and 100 rapid bootstraps. Species tree analyses were performed for all the gene trees with the program ASTRAL‐III 5.5.3 (Zhang et al., 2018). Low-supported clades (< 10%) were collapsed for the gene trees. Local posterior probabilities (LPPs) were estimated to provide support for clades, with LPP > 0.95 considered strongly supported (Mirarab et al., 2016).

The 130 plastid genes were trimmed with trimAl and then aligned with MAFFT version 7.407 (Katoh and Standley, 2013). We reconstructed a phylogenetic tree using RAxML version 8.2.12 based on the plastid data with the GTRGAMMA model and a rapid bootstrap algorithm (Stamatakis, 2014), as those used for the nuclear gene sequences.

### 2.3 Species network inference

The Species Networks applying Quartets (SNaQ) method (Solís-Lemus and Ané, 2016) was implemented in PhyloNetworks to explore potential hybridization events in *Vitis*. A total of 20 accessions were selected to represent *Vitis* and *Ampelocissus*. The RAxML gene trees for the 986 genes were used as an input and were summarized by quartet concordance factors. The fit of the models was tested, allowing a maximum of 0 to 8 reticulation events (*h*) and 25 independent runs, starting from the ASTRAL tree for the initial network (*h* = 0). For the subsequent *h*, the best network predicted by the previous *h* value was used as the next starting tree. The optimal network for each *h* value was selected by considering the highest log-likelihood value (Solís-Lemus and Ané, 2016) and evaluating the pseudolikelihood score profile of each *h*.

### 2.4 Herbarium morphological studies

We examined herbarium collections of BRIT, F, MEXU, and US (abbreviations following Thiers, 2020), as well as images of type specimens via JSTOR Global Plants (http://plants.jstor.org).

### 2.5 Conservation assessments

For any new species requiring conservation assessments, species distributions were evaluated using the Geospatial Conservation Assessment Tool GeoCAT (Bachman et al., 2011) to calculate the extent of occurrence (EOO) and the area of occupancy (AOO) for each species. ArcGIS maps of the World Database on Protected Areas (WDPA) (UNEP-WCMC, 2025) were utilized to determine *in situ* protection. Data from the distribution maps, EOO, AOO, counts of localities, and knowledge of habitat protection and land cover loss were combined to determine full conservation assessments using IUCN Red List categories and criteria (IUCN, 2012, 2022) for each species.

## 3 Results

### 3.1 Phylogenomic relationships inferred from nuclear data

**Figure 1:**
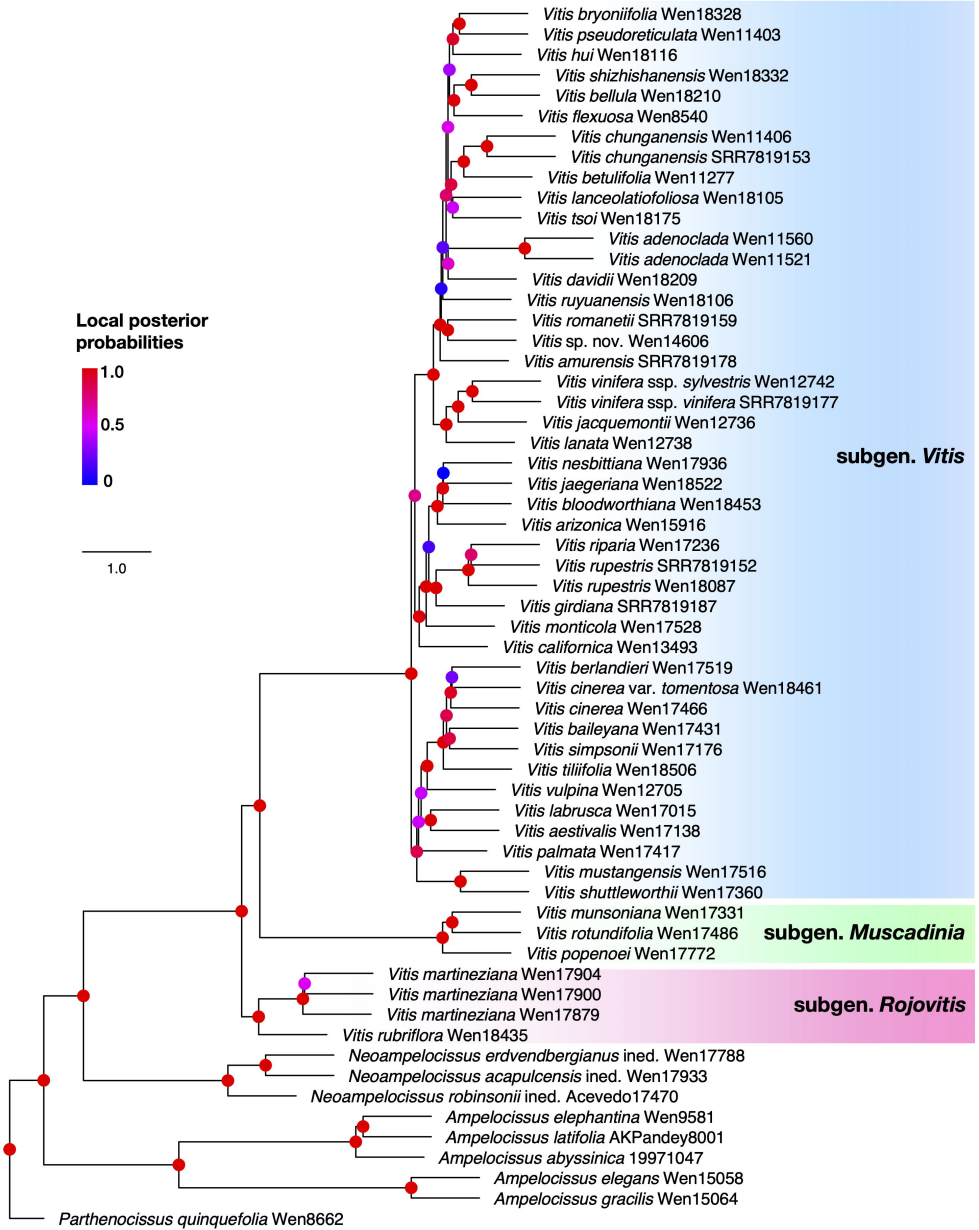
Astral tree of *Vitis* and its close relatives based on the nuclear data set. Branch support values (local posterior probabilities) are shown on nodes with colored circles. The three different subgenera are indicated in different colors, *Vitis* (blue), *Muscadinia* (green), and *Rojovitis* (pink).

The nuclear phylogenetic analyses were performed on 986 loci after excluding paralog genes. The coalescent-based ASTRAL species tree showed that the two morphologically unique new species, *Vitis martineziana* and *V. rubriflora*, form a clade sister to the clade of *Vitis* subgenus *Vitis* + subgenus *Muscadinia* (LPP = 1.00; **Figure 1**). *Vitis* subgenus *Vitis* is composed of members from Asia, Europe, and the New World and was well supported as a clade, which is sister to *Vitis* subgenus *Muscadenia*. The ML analysis based on the concatenated 986‐loci nuclear dataset using RAxML also resolved a well-supported phylogeny, with major nodes having 100% bootstrap support values (**Supplementary Figure S1**). All inferred nuclear phylogenetic trees depicted similarity with respect to the main clades (**Figure 1**; **Supplementary Figure S1**). However, there were several incongruences between the maximum likelihood and the coalescent trees within *Vitis* subgenus *Vitis*, such as the placements of *Vitis tiliifolia* and the *V. mustangensis-V. shuttleworthii* clade, which are not the focus of this paper (cf., **Figure 1**; **Supplementary Figure S1**).

### 3.2 Phylogenomic relationships based on plastome data

**Figure 2:**
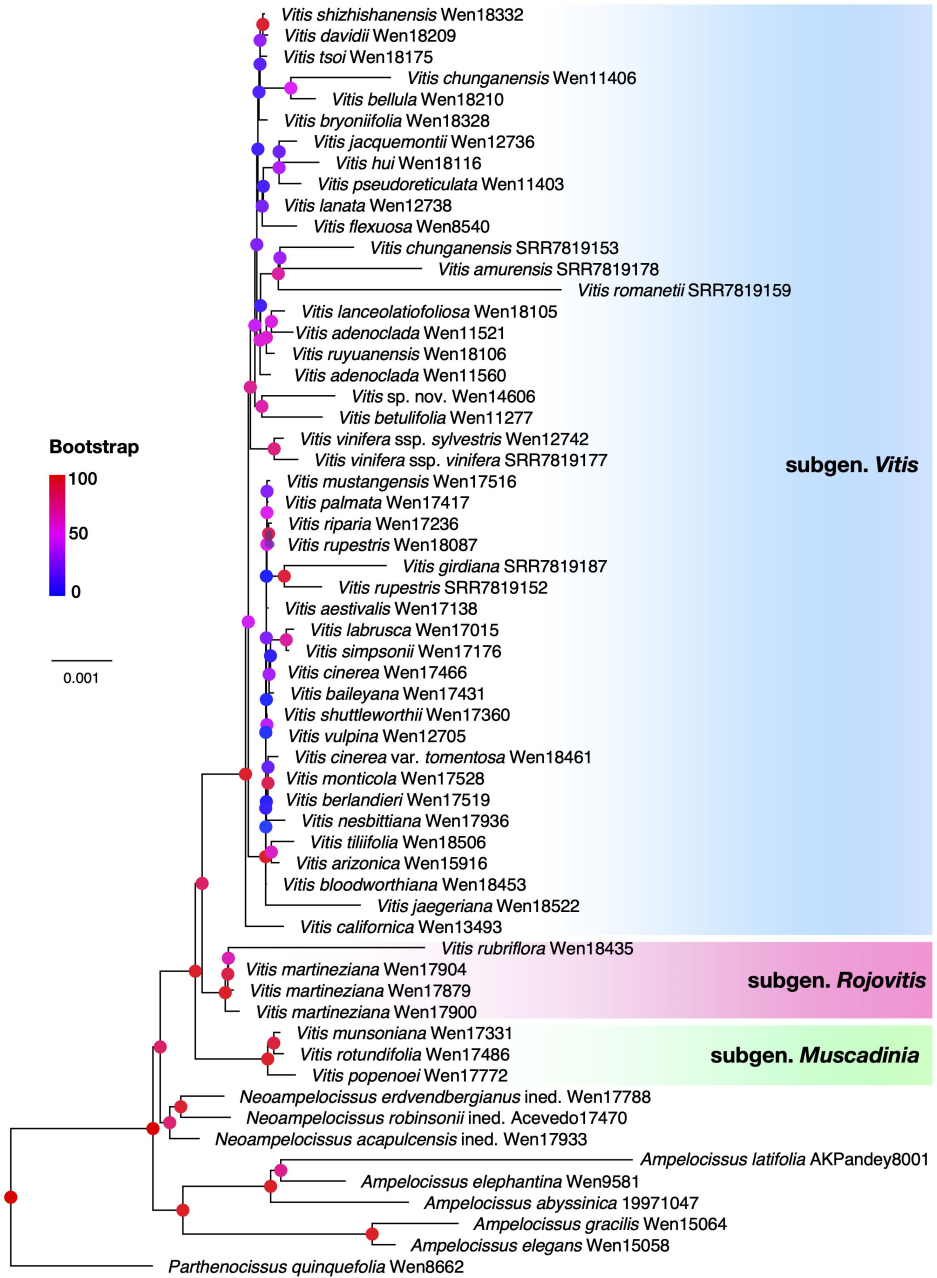
Maximum likelihood tree of *Vitis* and its close relatives based on 130 plastid coding sequences (CDS) genes. Branch support values (Bootstrap) are shown on nodes with colored circles. The three different subgenera are indicated in different colors, *Vitis* (blue), *Muscadinia* (green), and *Rojovitis* (pink).

**Figure 3:**
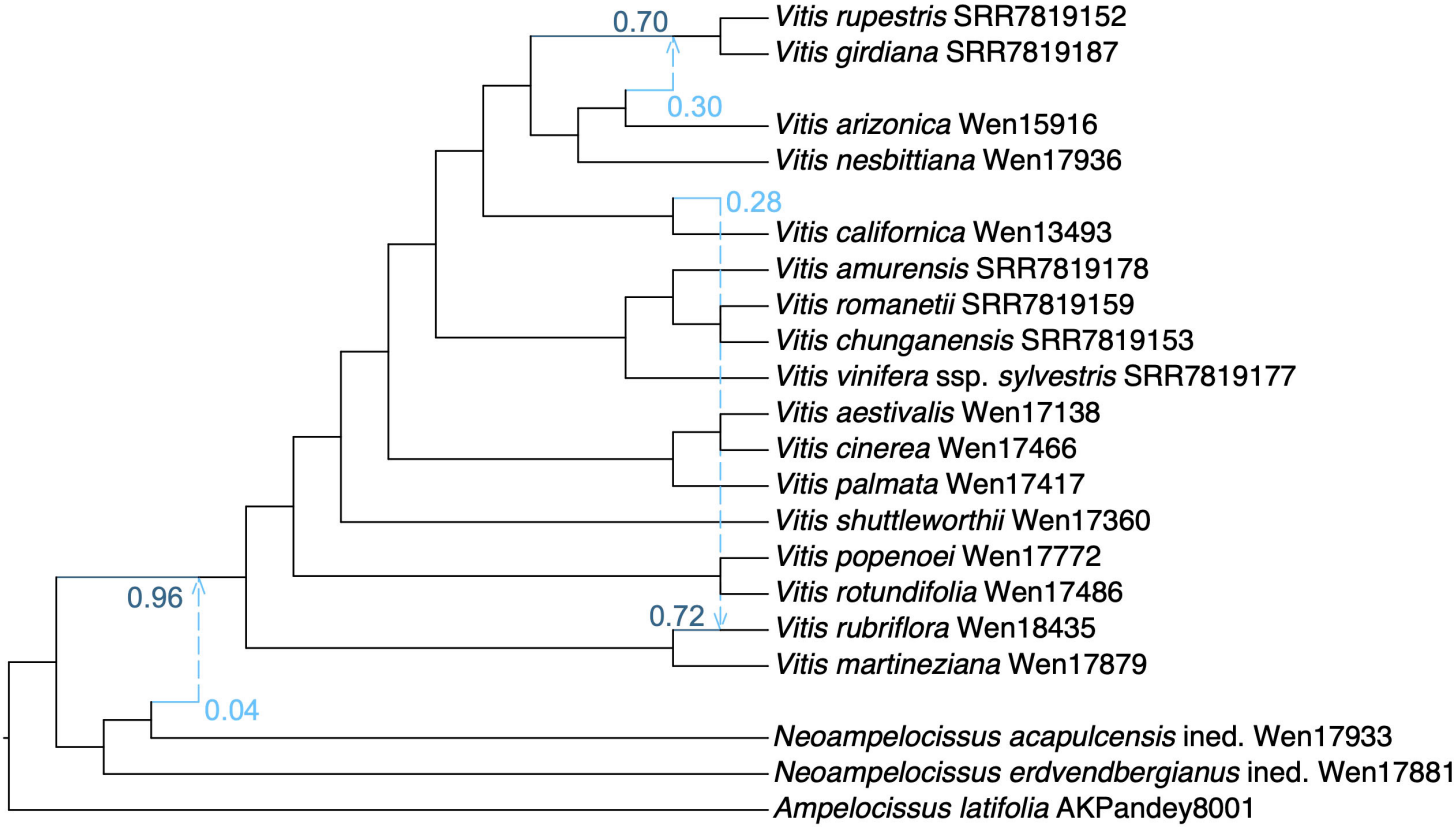
The optimal phylogenetic network inferred using the SNaQ/PhyloNetwork analysis with a representative sampling of *Vitis* and *Ampelocissus* (*h* = 3).

The ML analysis from plastome data (**Figure 2**) supported the *Rojovitis* clade sister to *Vitis* subgenus *Vitis*. *Vitis* subgenus *Muscadenia* is sister to the clade of *Vitis* subgenus *Vitis* + the *Rojovitis* clade. There are significant topological incongruences between the nuclear and plastid inferences within *Vitis* subgenus *Vitis* (cf. **Figures 2**, **3**; **Supplementary Figure S1**).

### 3.3 Species networks applying quartets

The SNaQ analysis (**Figure 3**) supported *h* = 3 as the optimal number of hybridization events inferred. The phylogenetic network results suggested a hybrid origin of *Vitis rubriflora* from Jalisco, with one parent being *V. martineziana* with the inheritance probability *γ* = 0.72, i.e., 72% of the *V. rubriflora* genome from *V. martineziana*. SNaQ showed the minor hybrid edge of *Vitis rubriflora* to be *V. californica*, now endemic to California and southern Oregon, with 28% genome contributions. The much smaller portion of the inheritance from *V. californica* suggested introgression.

## 4 Discussion

### 4.1 Discovery of a new subgenus *Rojovitis* of the grape genus

During our field studies in Mexico, we collected several morphologically unique specimens from Chiapas and Jalisco. These collections are highly distinctive in their lenticellate nonshredding bark, large loose paniculate thyrse inflorescence, and red flowers. Our phylogenetic analyses based on the nuclear and plastid data placed these specimens into a clade, with the nuclear species tree (**Figure 1**) showing the clade sister to the known species of *Vitis* and the plastid phylogeny (**Figure 2**) supporting the clade as sister to *Vitis* subgenus *Vitis*. We propose that the specimens of the clade from Chiapas and Jalisco represent a unique new subgenus, *Rojovitis*, of *Vitis*.

Our morphological studies group these specimens of the *Rojovitis* clade into two species based on the highly distinctive leaf morphology, and we describe them as two species new to science: *Vitis martineziana* J. Wen from Chiapas and *V*. *rubriflora* J. Wen from Jalisco. For nearly a century, two subgenera (*Vitis* and *Muscadinia*) have been recognized in *Vitis* (Rehder, 1927; Moore, 1991; Moore and Wen, 2016). The discovery of the third new subgenus of the economically important *Vitis* with two new species represents a major milestone of systematic research on grapes and their wild relatives.

The cytonuclear discordance supports a likely hybrid origin of *Vitis* subgenus *Rojovitis*, with an extinct lineage of *Vitis* subgenus *Vitis* as the maternal parent (**Figure 2**) and an early lineage of *Vitis* as the paternal parent (**Figure 1**). The results of the phylogenetic network analyses (**Figure 3**) suggest that *Vitis rubriflora* may represent an ancient hybrid species between *V. martineziana* and *V. californica*. The hybridization events concerning the evolution and diversification of *Vitis* subgenus *Rojovitis* will need to be further studied, with hybridization being shown to be an important mechanism in plant evolution and also in grape diversification (Harrison and Larson, 2014; Morales-Cruz et al., 2021; Ma et al., 2023; Nie et al., 2023).

### 4.2 Taxonomic treatment

***Vitis* subgenus *Rojovitis* J. Wen, subgen. nov.**

**Type species:** *Vitis martineziana* J. Wen.

**Diagnosis:** The new subgenus is highly distinctive from the other subgenera of the grape genus *Vitis* in its lenticellate, nonshredding bark; large, loose, paniculate thyrse inflorescences with well-developed secondary branches; and red, mostly bisexual flowers.

**Description:** High-climbing liana, andromonoecious; stem with prominent lenticels, bark not peeling. Tendrils two-forked, unequal, becoming stout. Leaves simple; blade ovate to broadly ovate, pubescent with both short simple and cobwebby hairs on the abaxial surface; base sagittate to V-shaped; margin serrate or serrulate. Inflorescence is a large, loose, paniculate thyrse with well-developed secondary branches. Flowers are five-merous, mostly bisexual; petals red, coherent by margins; floral disc sulcate. Fruits globose.

It contains two species: *Vitis martineziana* J. Wen, endemic to Chiapas, and *V. rubriflora* J. Wen, from Jalisco—both new to science.

**Figure 4:**
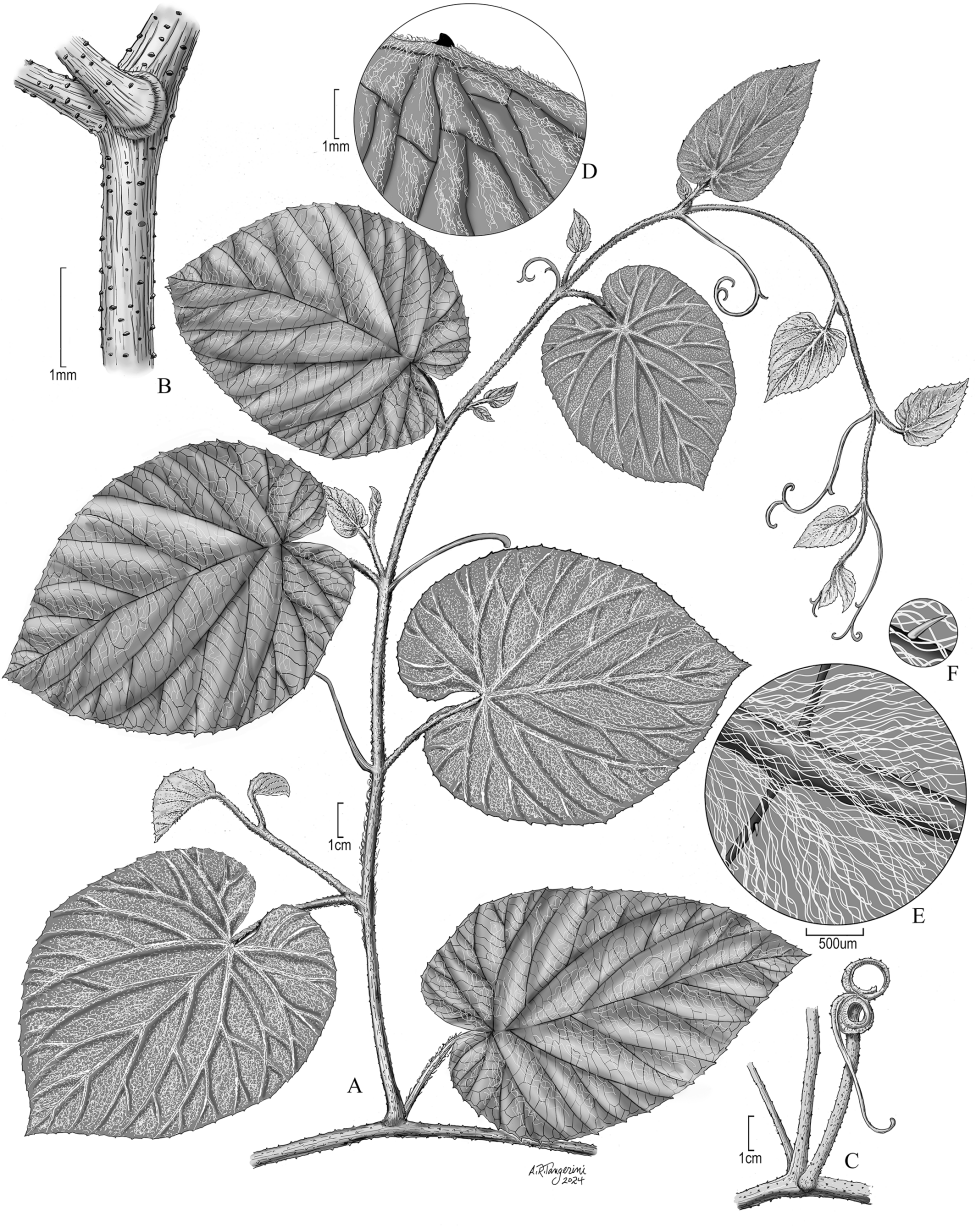
Vegetative characters of *Vitis martineziana* J. Wen. **(A)** A branch showing vegetative stem, tendril, and leaf morphology (*J. Wen 8704*, US). **(B)** Stem showing lenticels, an old tendril base on the right, and a branch on the left. **(C)** An old woody tendril on the right, a branch in the middle, and a petiole subtending the branch on the left. **(D)** Leaf adaxial (upper) surface. **(E)** Leaf abaxial (lower) surface. **(F)** Leaf abaxial surface showing the simple and cobwebby hairs. **(B–F)** Based on J. *Wen 17884* (US).

**Figure 5:**
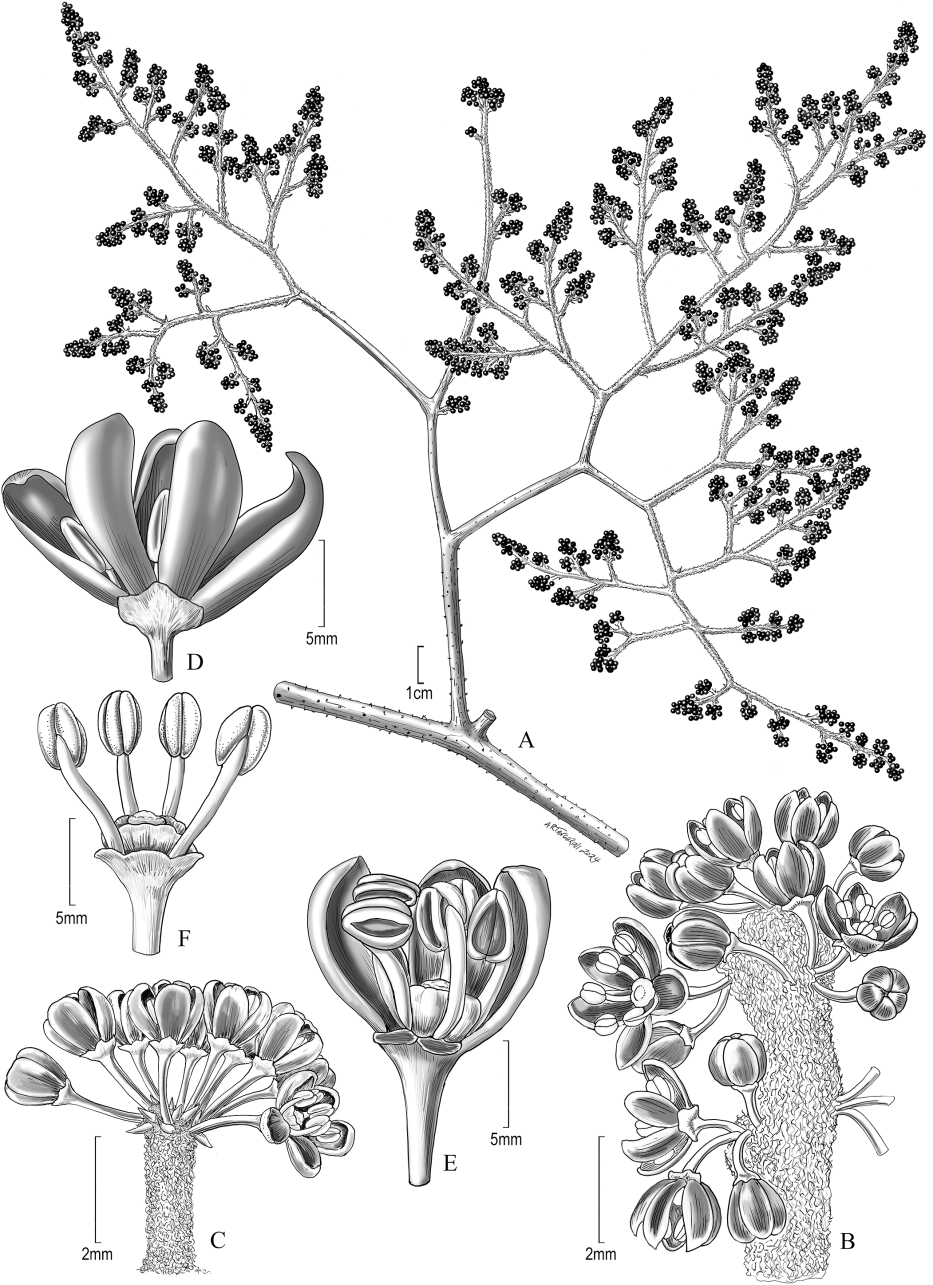
Reproductive characters of *Vitis martineziana* J. Wen. **(A)** Inflorescence morphology (*D.E. Breedlove 9050*, US). **(B)** Inflorescence unit. **(C)** Another inflorescence unit, more umbellate. **(D)** Opening flower. **(E)** Opening flower with two petals removed to show stamens, stigma, and floral disc. **(F)** An older flower with all petals removed. **(B–F)** Based on *D.E. Breedlove 30406* (BRIT).

***Vitis martineziana* J. Wen, sp. nov. (** **Figures 4**, **5**)

**Type:** Mexico: Chiapas, Municipio Tuxtla Gutiérrez, steep slope along the road to El Sumidero de Tuxtla, 20 km north of Tuxtla Gutiérrez, elev. 4,300 ft, 16 February 1965, fl, *D.E. Breedlove 9050* (holotype: US!, 03373396, inflorescence; isotype: F!, 1624629).

**Diagnosis:** Compared to *Vitis rubriflora*, *V*. *martineziana* possesses chartaceous mature leaves; its adaxial surface is sparsely pubescent with cobwebby long hairs, while its abaxial surface is tomentose with short and cobwebby long hairs. The leaf margin serrulate.

**Description:** Liana, andromonoecious; stems cobwebby to glabrescent, with lenticels; bark not peeling. Tendrils two-forked, becoming woody and stout. Stipules 2–3 mm, triangular. Leaves simple, chartaceous, petioles 3–5 cm; blade ovate to broadly ovate, 11–25 cm × 8–28 cm, adaxial surface sparsely pubescent with cobwebby hairs, often with visible raphides, rarely with simple short hairs; abaxial surface tomentose with cobwebby long hairs and simple short hairs; base sagittate to cordate, sometimes overlapping; margin serrate; apex acute to acuminate. Inflorescence is a thyrse with long lateral branches, appearing before leaves; peduncles densely cobwebby; pedicels 2–3 mm, glabrous. Flowers red; calyx truncate, glabrous; petals 5, 2.5–3 mm × 0.8–1 mm, elliptic, coherent by margins, caducous, glabrous; stamens 5, 2–2.5 mm, anthers 0.5 mm long; disc adherent to the ovary, five-sulcate, ovary apex red, glabrous; style obsolete; stigma punctate. Fruits globose, 10–11 mm in diameter.

**Etymology:** The new species is named in honor of Mr. Esteban Manuel Martínez Salas, a botanist in the Herbarium, Instituto de Biología, Universidad Nacional Autónoma de México (MEXU) and arguably the most prolific Mexican plant collector.

**Figure 6:**
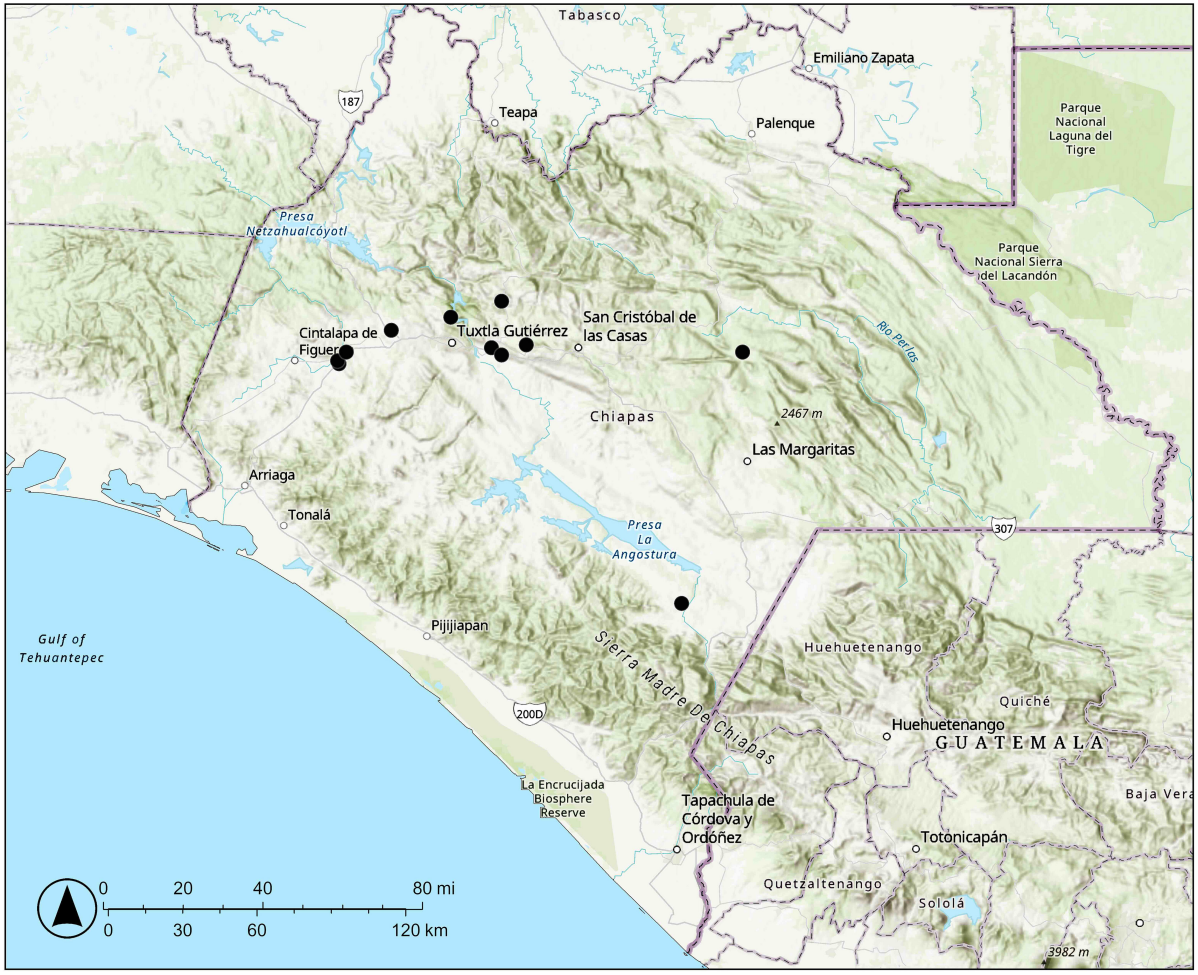
Distribution of *Vitis martineziana* J. Wen in Chiapas, Mexico.

**Distribution and ecology:** Found in Chiapas, Mexico (**Figure 6**); occurs in tropical montane deciduous or semideciduous forests, in limestone habitats; at elevations of 470–1,500 m.

**Conservation:** *Vitis martineziana* has an extent of occurrence (EOO) of 10,287 km^2^ and an area of occupancy (AOO), measured using a 2 × 2 km grid, of 40 km^2^. There are eight known localities. While one locality exists within the Sumidero Canyon National Park, the others are in landscapes experiencing habitat destruction and degradation, mainly due to urban expansion, extensive agriculture, and extensive livestock farming. Climate change is predicted to have a high impact on this region (Esperon-Rodriguez et al., 2019) and may further threaten the survival of this species. *Vitis martineziana* is extremely rare. During a 2022 survey, three or fewer mature individuals were recorded at each locality, suggesting that fewer than 50 total mature individuals remain (J. Wen, pers. obs.). The species is not found in cultivation and is not known from any *ex-situ* collections. With fewer than 50 total mature individuals, *Vitis martineziana* is preliminarily assessed as critically endangered (CR) under IUCN Red List criterion D.

**Additional specimens examined:** Mexico. Chiapas: Avispero, Comitán to Motozintla, 530 m, 15 April 1946, fruit with emerging leaves, *E. Hernández Xolocotzi X-1398* (MEXU). Municipio de Berriozábal, 5 km east of Berriozábal along Mexican Highway 190, gentle slope with tropical deciduous forest, *Bursera*, *Ceiba*, and *Heliocarpus*, elev. 800 m, 17 December 1972, flowers red, *D.E. Breedlove & R.F. Thorne 30406* (BRIT). Municipio Bochil, along Rt. 195, 5 km N of Soyalo, 16° 54.27′N, 092° 55.54′W, 1,483 m, climber on shrubby slope, 15 May 2006, *J. Wen 8708* (MEXU, US); along Rt 195, 5 km N of Soyalo, N16° 54.322′, W92° 55.699′, 4,790 ft, in tropical deciduous forest, slender climber, 19 August 2022, *J. Wen 17900* (MEXU, US). Municipio Chiapa de Corzo, 9 km E of Chiapa de Corzo, El Chorreadero, along Mexico Hwy. 190, roadside disturbed area, 16° 44.903′N, 092° 50.094′W, 688 m, 15 May 2006, *J. Wen 8704* (MEXU, US); El Chorreadero, 5.6 miles east of Chiapa de Corzo along Mexican Highway 190, elev. 2,500 ft, 24 February 1966, fl, *R.M. Laughlin 193* (MEXU); along old Rt. 190, 9 km E of Chiapa de Corzo, 16° 44′19″N, 92° 57′56″W, 471 m elev., tropical semideciduous forest, limestone area, 20 August 2022, *J. Wen 17904* (MEXU, US). Municipio Ocozocoautla, El Palmar, along Rt. 190, between Km markers 103 and 104, near junction with the road to San Jorge, 16° 43′8″N, 93° 1′4″W, 735 m elev., 18 August 2022, stem, tendril, and leaves only, *J. Wen 17883* (MEXU, US, two sheets). Municipio Ocozocoautla, El Yeso, near jct. of Rt. 190 and unpaved road to the gypsum mine, 16° 40.853′N, 093° 32.546′W, 620 m, in disturbed tropical dry forest, climber on fence, 15 May 2006, *J. Wen 8697* (MEXU, US); Municipio Ocozocuautla, km 18 Racho, along Rt. 190, between km markers 103 and 104, El Yeso, near jct. of Rt. 190 and unpaved road to the gypsum mine, 16° 43.132′N, 093° 31.075′W, in a disturbed area with limestone bedrock, fruits reddish, *J. Wen 8702* (MEXU, US); El Yeso, along Rt. 190, near km marker 95–96, by Rancho de el Yeso, N16° 41′19″, W93° 32′48″, 574 m, 18 August 2022, *J. Wen 17879* (MEXU, US).

Lombardi (2005) described *Ampelocissus mesoamericana* (**Supplementary Figure S2**) from Central America and noted that *Ampelocissus mesoamericana* is characterized by the presence of lenticels on the stem and the obpyriform floral buds. Lombardi (2005) designated the holotype specimen—*J. M. Tucker 905* from El Salvador—preserved at the US National Herbarium (US) in Washington, DC. However, the holotype appears to represent a species closely related to, if not identical, *Ampelocissus erdvendbergianus*, which lacks lenticels on the stem and has a hirsute leaf margin and three-branched tendrils. The specimens cited in Lombardi’s *Ampelocissus mesoamericana* belong to two species, with the holotype likely representing *Ampelocissus erdvendbergianus*.

**Figure 7:**
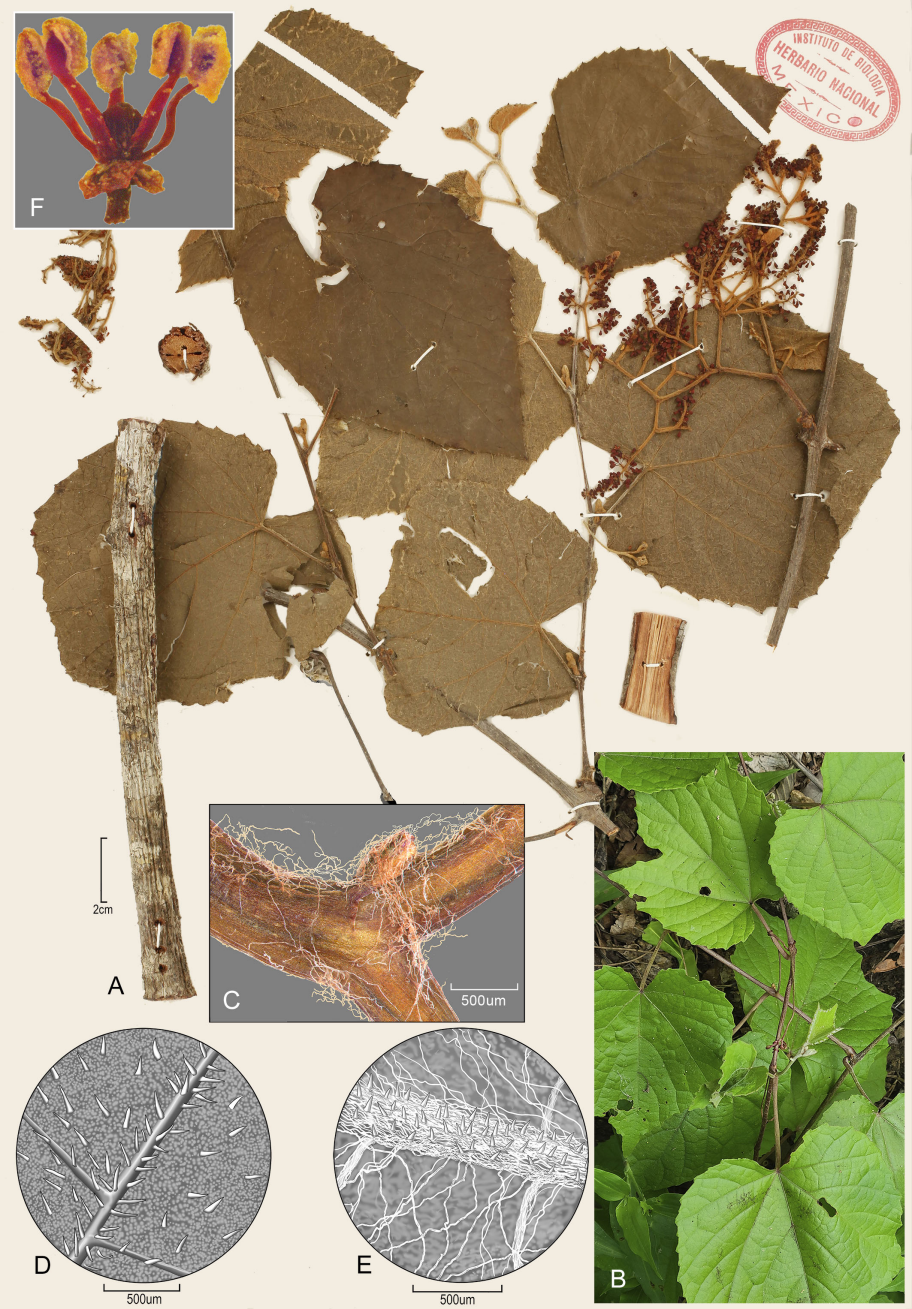
*Vitis rubriflora* J. Wen. **(A)** The holotype specimen showing leaves, inflorescence, and stem morphology. **(B)** A branch of a juvenile specimen, *Wen 18435* (US). **(C)** Cobwebby pubescence on the tendril branches, *Wen 18437* (US). **(D)** Adaxial (upper) leaf surface showing short hairs, Wen *18435* (US). **(E)** Abaxial (lower) leaf surface showing both short and cobwebby hairs, *Wen 18435* (US). **(F)** Flower after falling off of the petals (*J*. *Arburo S. Magallanes 3983*, MEXU).

***Vitis rubriflora* J. Wen, sp. nov.** (**Figure 7**).

**Type:** Mexico: Jalisco: Municipio La Huerta, Estación de Investigación, Experimentación y Difusión Chamela, UNAM, Trail Tejón, c. 3,000 m away from its trail start, no elevation indicated, selva baja caducifolia, bejuco, flor roja, 4 February 1983, fl, with leaves, *J. Arturo S. Magallanes 3983* (Holotype: MEXU; isotype: EBCH).

**Diagnosis:** In comparison with *Vitis martineziana*, *V. rubriflora* has thin, papery mature leaves; its adaxial surface is sparsely pilose with short hairs on veins and veinlets, and its abaxial surface is sparsely pubescent with short and cobwebby long hairs; the leaf margin is serrate.

**Description:** Liana, andromonoecious; stem with light reddish wood; bark not shredding, lenticellate, with vertical grooves with age, young branches slender, pubescent with whitish hairs. Tendrils biforked. Leaves ovate, not lobed at maturity, three-lobed on juvenile branches, lateral veins five to six on each side; margin finely serrate; apex acuminate to acute; base V-shaped; adaxial surface pilose with short hairs; abaxial surface with two types of hairs—pilose with short hairs mostly on veins and veinlets, and sparsely cobwebby with long hairs throughout; tomentose when young at the shoot apex. Inflorescence a paniculate thyrse, 10–20 cm long, densely cobwebby-tomentose; pedicels 2.5–3.5 mm, glabrous. Flowers red; calyx with 5-min teeth, glabrous; petals 5, 2–2.5 mm × 0.7–0.9 mm, elliptic, mid-vein conspicuous, coherent by margins, caducous on male flowers, glabrous; stamens 5, 2.2–2.6 mm, anthers 0.5 mm long, disc adherent to ovary, five-sulcate, ovary glabrous, style obsolete, stigma punctate. Young fruits are globose, based on residue petals and stamens, mature fruits are not seen.

**Figure 8:**
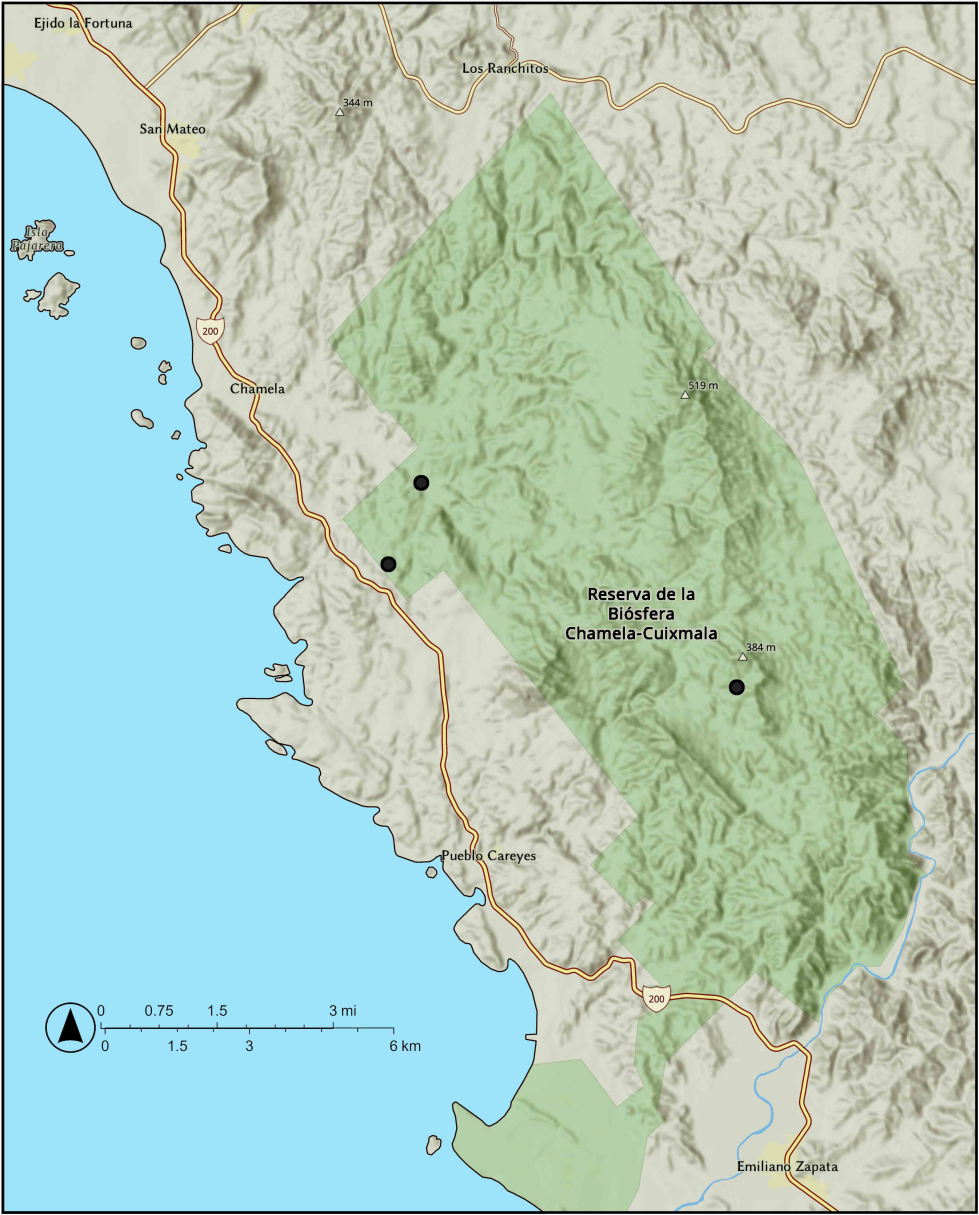
Distribution of *Vitis rubriflora* J. Wen in Chamela, Jalisco, Mexico.

**Distribution and ecology:** The species is very rare and is only known from the Chamela area in Municipio La Huerta, Jalisco, Mexico (**Figure 8**). It occurs in tropical lowland deciduous forests near the sea level.

**Conservation:** *Vitis rubriflora* has an EOO of 7.04 km^2^ and an AOO, as measured with a 2 × 2 grid, of 12 km^2^. There are two known localities, both situated within the Chamela-Cuixmala Biosphere Reserve. Although the habitat is protected, its management—including weed removal and trail maintenance—affects the species. Furthermore, the species may be impacted by extreme weather events. Research on bioclimatic variables in the Chamela-Cuixmala Biosphere Reserve indicates increased vulnerability to climate change (Esperon-Rodriguez et al., 2019), along with an annual rise in temperature and greater precipitation during the wet season, associated with an increase in the number of large storms (Takano-Rojas et al., 2023). These changes put the species at risk from hurricane-force winds and flash floods. *Vitis rubriflora* is also extremely rare. During a 2024 survey, only two individuals were recorded, and it is estimated that fewer than 10 mature individuals remain in the wild (J. Wen, pers. obs.). It is not found in cultivation, and it is not known from any *ex-situ* collections. With fewer than 10 total mature individuals, *Vitis rubriflora* is preliminarily assessed as CR under IUCN Red List criterion D.

**Additional specimens examined:** Mexico. Jalisco: Municipio La Huerta, Chamela Bay region, Rancho Cuixmala, “Cumbres de Cuixmala”, 19° 29′N, 104° 58′W, tropical deciduous forest, vine, inflorescence red, stamens white, young fruits reddish green, 19 April 1991, fl, without leaves, *M. G. Ayala 91-55* (BRIT); Municipio La Huerta, Chamela, UNAM Estación de Biología.19° 30′ 47.8″N, 105° 02′ 14.8″W, elevation at 25 m, *J. Wen* et al.*, 18435* (US, MEXU); same location, *J. Wen* et al.*, 18437* (US, MEXU).

**Taxonomic key to species of *Vitis* subgenus *Rojovitis*:**

1. Mature leaves chartaceous; adaxial surface sparsely pubescent with cobwebby long hairs; abaxial surface tomentose with short and cobwebby long hairs; leaf margin serrulate............................................*Vitis martineziana*
1. Mature leaves thin and papery; adaxial surface sparsely pilose with short hairs on veins and veinlets; abaxial surface sparsely pubescent with short and cobwebby long hairs; leaf margin serrate...................................*Vitis rubriflora*

**Taxonomic key to the three subgenera of *Vitis*:**

1. Flowers are mostly bisexual, petals red; inflorescences loosely paniculate thyrses; in tropical dry deciduous forests.............................................................Subgenus *Rojovitis*
1. Flowers mostly unisexual; petals greenish white; inflorescences compact thyrses; in temperate to tropical mesic forests..................................................................................2
2. Tendrils simple; bark adherent with prominent lenticels; pith continuous through nodes...........Subgenus *Muscadinia*
2. Tendrils bifid to trifid, rarely simple; bark shedding, the lenticels inconspicuous; pith interrupted by diaphragms at nodes......................................................................Subgenus *Vitis*

This discovery of a new subgenus with two new species in the economically important grape genus showcases that biodiversity discovery is far from complete today. The biodiversity community needs to emphasize field exploration, especially in poorly collected regions, in the new age of discovery (Wen et al., 2023). The grape industry has heavily emphasized the utilization of *Vitis vinifera* from Eurasia; however, the adaptability of the industry to climate change and pests depends on further exploration of resources within the grape genus *Vitis*. As the early-diverged taxa in *Vitis* may also have involved hybridizations (**Figure 1**), these newly discovered germplasm resources could benefit humanity and potentially serve as important models for studying adaptation and character evolution. These species, however, urgently need to be protected and properly managed due to extensive habitat loss and alteration.

## Data Availability

The datasets presented in this study can be found in online repositories. The names of the repository/repositories and accession number(s) can be found in the article/**Supplementary Material**.

## Supplementary material

Supplementary Figure 1 Maximum likelihood tree of *Vitis* based on 986 concatenated nuclear genes. Branch support values (Bootstrap) on nodes are displayed with colored circles. The three different subgenera are indicated in different colors, *Vitis* (blue), *Muscadinia* (green) and *Rojovitis* (pink).

Supplementary Figure 2 Holotype of *Ampelocissus mesoamericana*.
