# Supplementary figures and images for: Phylogenomic analyses support a new subgenus, Rojovitis, of the grape genus Vitis from Mexico
Source: Front Plant Sci. 2025 May 19;16:1580648. doi: 10.3389/fpls.2025.1580648 (PMC12127385; doi:10.3389/fpls.2025.1580648)

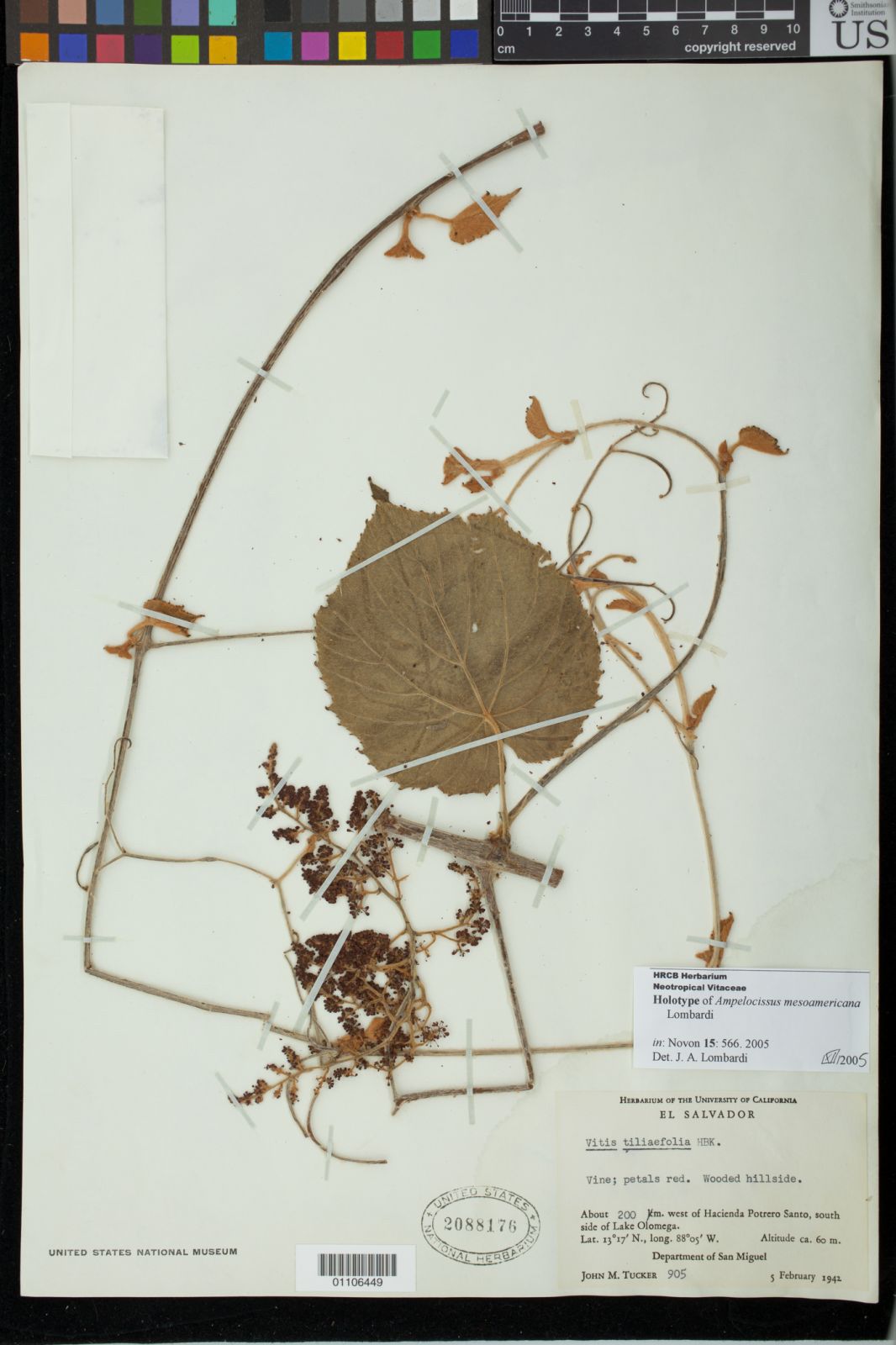

Supplement: Supplementary Figure 1 — Maximum likelihood tree of Vitis based on 986 concatenated nuclear genes. Branch support values (Bootstrap) on nodes are displayed with colored circles. The three different subgenera are indicated in different colors, Vitis (blue), Muscadinia (green) and Rojovitis (pink). [file Image1.jpeg]

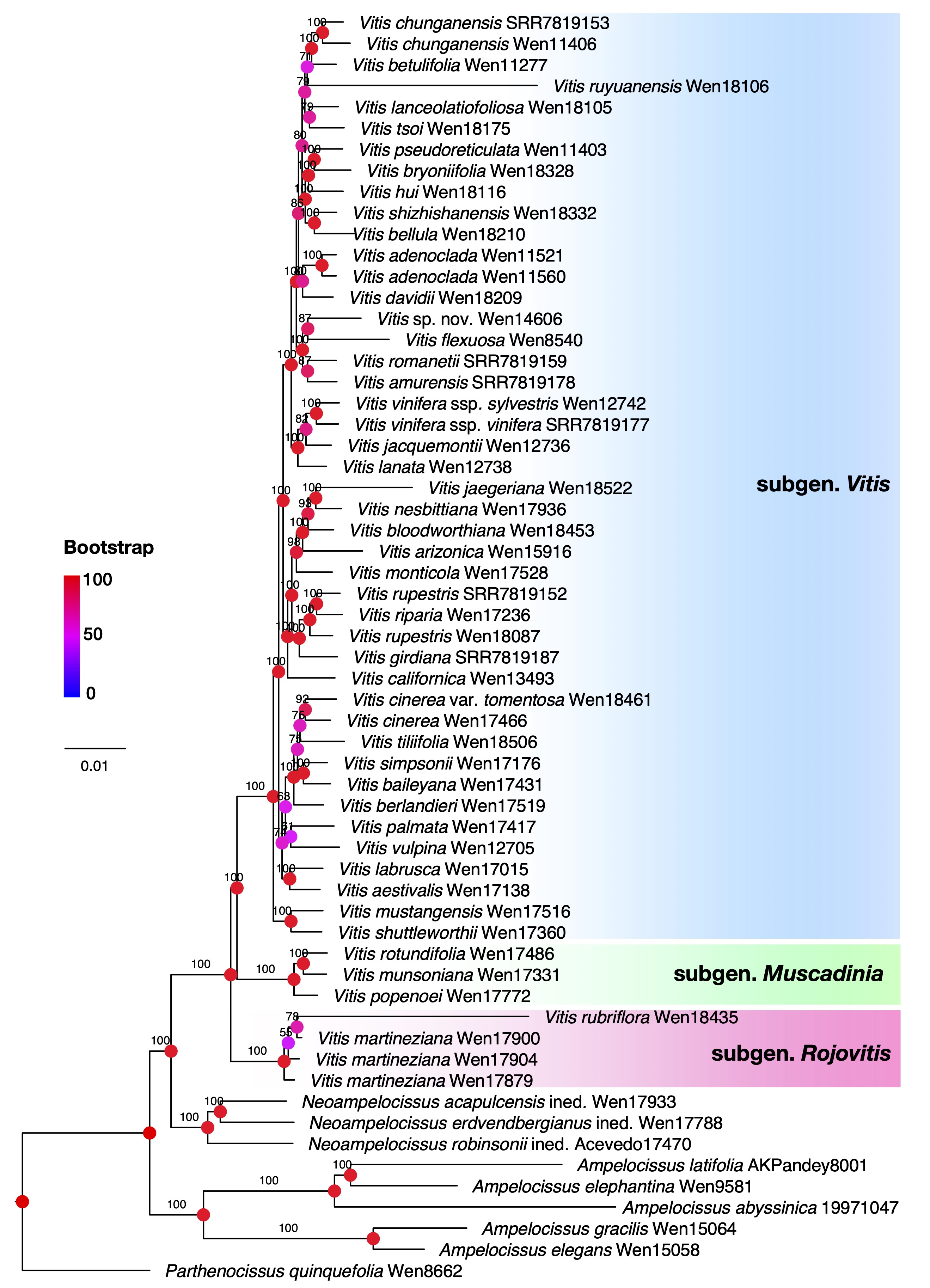

Supplement: Supplementary Figure 2 — Holotype of Ampelocissus mesoamericana. [file Image2.jpeg]
